# Role of subtyping in detecting *Salmonella *cross contamination in the laboratory

**DOI:** 10.1186/1471-2180-9-155

**Published:** 2009-07-31

**Authors:** Niall De Lappe, Jean O Connor, Geraldine Doran, Genevieve Devane, Martin Cormican

**Affiliations:** 1 National Salmonella Reference Laboratory, Department of Medical Microbiology, Galway University Hospitals, Galway, Ireland; 2 Department of Bacteriology, National University of Ireland, Galway, Galway, Ireland

## Abstract

**Background:**

With the exception of *M. tuberculosis*, little has been published on the problems of cross-contamination in bacteriology laboratories. We performed a retrospective analysis of subtyping data from the National *Salmonella *Reference Laboratory (Ireland) from 2000–2007 to identify likely incidents of laboratory cross contamination.

**Methods:**

Serotyping and antimicrobial susceptibility testing was performed on all *Salmonella *isolates received in the NSRL. Phage typing was performed on all *S*. Typhimurium and *S*. Enteritidis isolates while multi-locus variance analysis (MLVA) was performed on selected *S*. Typhimurium isolates. Pulsed field gel electrophoresis (PFGE) using the PulseNet standard protocol was performed on selected isolates of various serovars.

**Results:**

Twenty-three incidents involving fifty-six isolates were identified as likely to represent cross contamination. The probable sources of contamination identified were the laboratory positive control isolate (n = 13), other test isolates (n = 9) or proficiency test samples (n = 1).

**Conclusion:**

The scale of laboratory cross-contamination in bacteriology is most likely under recognized. Testing laboratories should be aware of the potential for cross-contamination, regularly review protocols to minimize its occurrence and consider it as a possibility when unexpected results are obtained.

## Background

Laboratory contamination can be defined as the inadvertent addition of analytes to test samples during sample collection, transportation or analysis. There is a high level of awareness of the potential for cross contamination when using nucleic acid amplification methods [1]. Although conventional microbial culture also represents amplification of signal to detectable levels there is relatively little systematic data on the frequency of cross contamination in conventional microbiology. In clinical laboratories cross contamination can lead to misdiagnosis of patients, inappropriate treatment or isolation of patients and investigation of pseudo-outbreaks. Detection of pathogens in food items can lead to very significant economic loss [2] therefore it is important to ensure that positive results reflect true product contamination.

Sources of microbial laboratory contamination may include positive control strains, cultures of recent isolates, laboratory workers and airborne exogenous material such as fungal spores. Pseudo-outbreaks due to cross-contamination of patient samples have been reported with *Aspergillus niger *[3] and Vancomycin Resistant Enterococci (VRE) [4] however most of the existing literature relates to *M. tuberculosis*. Various studies have shown that the rates of false positive results due to cross-contamination by *M. tuberculosis *varies from 0.33 to 8.6% [5] with contamination reported to occur most commonly during the initial processing of specimens [6]. The change in use from solid media to more sensitive, automated broth cultures has increased sensitivity and shortened the time to detection but has also led to increased numbers of false positives [5]. Other factors reported to be responsible for contamination include clerical errors, spillages and splashes, aerosol formation [7], contamination of equipment used to dispense reagents [8], use of automatic pipettes [9], and new or poorly trained staff.

Laboratory cross contamination is more likely to be suspected in the context of a series of isolates of an uncommon strain clustered in time. In the case of commonly isolated bacteria sporadic or intermittent contamination may be entirely unsuspected. For example isolation of *Staphylococcus aureus *or *Salmonella enterica *from 2 or more specimens in a short period of time is not an uncommon event. In the absence of detailed subtyping of common species to allow recognition of relationships between isolates cross contamination may go undetected. As a result of detailed sub-typing of *Salmonella enterica *isolates and liaison with service users we became aware of a number of incidents of probable laboratory cross contamination. Here we present a review of our data and records of liaison over a period of 8 years to emphasise the scale of this problem and the role of reference laboratories in detection and investigation of suspected laboratory contamination.

## Results

### Summary of Results

Twenty-three incidents of probable laboratory cross contamination involving fifty-six isolates were identified. Food laboratories accounted for the majority of incidents (n = 20) with just 3 incidents associated with human clinical samples. Contamination with the laboratory positive control isolate accounted for the majority of suspected incidents (n = 13) while contamination with other test isolates (n = 9) or proficiency test samples (n = 1) accounted for the remainder (Additional file 1). Two specific food laboratories accounted for 4 contamination incidents each. MLVA proved a useful technique in detection of incidents involving *S*. Typhimurium (Table 1). The use of 5 separate loci for PCR amplification gives an allele string which results in good discrimination, even among closely related isolates.

**Table 1:** Case 3 – Molecular Analysis of *S*. Typhimurium PT Untypable, ASSuT isolates in NSRL databases.

Isolate no	Year	Lab	Source	PFGE		MLVA
				XbaI	BlnI	
03–0407	2003	D	Human	B	A	05-02-07-14-02
05–0802	2005	E	Human	A	A	04-03-10-02-02
05–0900	2005	E	Dairy product	B	A	04-04-11-00-02
05–0902	2005	E	Swine	B	A	04-04-11-00-02
07–0146	2007	E	Dairy product	A	B	04-03-11-02-02
07–0237	2007	E	Swine	A	B	04-03-11-02-02
07–0200	2007	L	Pork	C	C	05-02-07-00-02
07–0201	2007	L	Unknown	B	A	04-03-07-02-02
07–0204	2007	L	Unknown	A	A	04-04-16-21-02
07–0028	2007	L	Pork	A	A	04-03-10-02-02
07–0060	2007	L	Pork	A	A	04-03-10-02-02
07–0174	2007	L	Swine	A	A	04-03-11-02-02

Investigation of a suspected contamination incident involving 07–0146, a *S*. Typhimurium, PT Untypable, resistance profile ASSuT, isolated from a dairy product involved molecular analysis of all isolates sharing this isolates phenotype (n = 12). PFGE with XbaI digestion showed the isolates to be closely related, e.g. patterns A and B were 92.8% similar while C was 89% similar to A. All isolates were indistinguishable with BlnI digestion apart from 07–0146 and 07–0237 (86% similarity) and 07–0200. MLVA provided further evidence that the *Salmonella *isolated from the dairy product was in fact contamination from swine isolate 07–0237.

The 2005 Lab E dairy isolate (05–0900) differed from 07–0146 but was indistinguishable from a swine isolate (05–0902) from Lab E which was isolated at the same time.

Below is a description of 3 of the 23 incidents.

### Case 1

A review of our databases showed that from October 2003 to April 2004 11/30 (37%) of isolates received from an accredited private food laboratory (Lab A) were identified as *S*. Typhimurium DT132 (Additional file 1). The isolates were stated to have originated from unrelated food products including beef (n = 7), pork (n = 2), a drain swab (n = 1) and powder (n = 1). When submitted the laboratory quality control strain was also *S*. Typhimurium DT132. Following discussion with the sending laboratory no further *S*. Typhimurium DT132 isolates were received from this laboratory.

### Case 2

This incident occurred in the Clinical Microbiology department of a teaching hospital (Lab C) [10]. A stool sample from a 78 year old female patient was submitted for analysis. No colonies resembling *Salmonella *were observed on the primary culture plates however *Salmonella *was isolated on day two following subculture of the selenite broth to xylose lysine deoxycholate (XLD) agar. The isolate was typed as *S*. Enteritidis PT1, with resistance to nalidixic acid. Another *S*. Enteritidis PT1 with resistance to nalidixic acid was isolated during the same 2 day period in the same laboratory from a female patient with a history of profuse diarrhoea associated with travel outside of Ireland and requiring hospital admission. The 78 year old female patient had been a hospital inpatient on naso-gastric feeding for an extended period prior to isolation of *Salmonella*. The clinical history was of a brief episode of loose stool and all subsequent specimens were negative for *Salmonella*.

### Case 3

An accredited private food laboratory (Lab E) submitted an isolate (07–0146) of *Salmonella *stated to have been isolated from a dairy product (Additional file 1). The laboratory had been testing swine samples at the time of this isolation and suspected cross-contamination. The isolate typed as *S*. Typhimurium, was untypable by phage typing, i.e. did not react with any of the typing phages, and was resistant to ampicillin, streptomycin, sulphonamide and tetracycline (ASSuT). A literature review showed that this phenotype was associated with swine [11]. As part of the investigation we asked the laboratory to forward all their group B *Salmonella *isolates (n = 51) from that year for typing. Serotyping divided these isolates into 6 different serotypes including 17 *S*. Typhimurium isolates. Phage typing and antimicrobial susceptibility testing subdivided the 17 *S*. Typhimurium isolates into 10 phenotypes, of which a single isolate, 07–0237, matched 07–0146, i.e. phage untypable and ASSuT resistance. This isolate from pork predated the isolate from the dairy product and we suspected this to be the source of contamination.

We searched our databases since 2000 and identified 10 additional isolates with this phenotype. These included 2 human faecal isolates, 2 from unknown food sources, 5 from porcine sources and an isolate from a dairy product from 2005 from the same laboratory involved in this incident (Table 1). We performed molecular subtyping on these isolates to determine the likelihood of their having coming from the same source.

**Figure 1:**
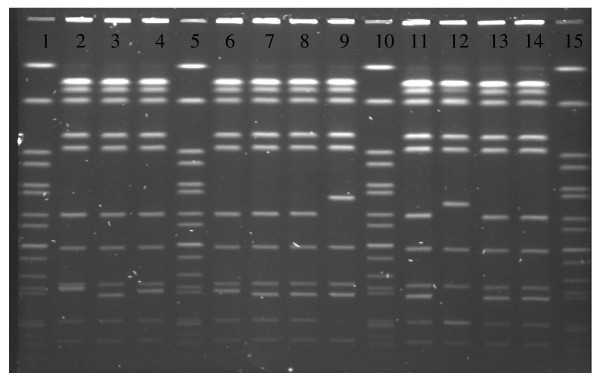
**Pulsed-field gel electrophoresis (PFGE) profiles of representative *S*. Typhimurium, PT Untypable, ASSuT isolates digested with BlnI**. Lane 1, H9812 (*S*. Braenderup control), lane 2, 03–0407, lane 3, 05–0802, lane 4, 05–0900, lane 5, H9812 (*S*. Braenderup control), lane 6, 05–0902, lane 7, 07–0028, lane 8, 07–0060, lane 9, 07–0146, lane 10, H9812 (*S*. Braenderup control), lane 11, 07–0174, lane 12, 07–0200, lane 13, 07–0201, lane 14, 07–0204, lane 15, H9812 (*S*. Braenderup control). PFGE with both XbaI and BlnI was performed on all isolates with same phenotype as isolate 07–0146. Digestion with BlnI proved more discriminatory showing 07–0146 and 07–0237 to be indistinguishable from each other and different from other isolates in our collection.

PFGE using XbaI showed most of the isolates to be closely related. However digestion with BlnI differentiated 07–0146 (Figure 1) and 07–0237 (data not shown) from the other isolates. MLVA separated the 12 isolates into 7 types (Table 1). Isolates 07–0146 and 07–0237 and a third recent porcine isolate from another laboratory were indistinguishable by MLVA. This group of 3 isolates were distinguishable from the remaining 9 isolates with the shared phenotype. This provided further proof that the isolation of 07–0146 from the dairy product resulted from a laboratory contamination incident.

## Discussion

There is very general recognition of the risk of laboratory cross contamination in nucleic acid amplification assays. Although airborne molecular contamination is one possibility contamination may also be as a result of direct or indirect contact contamination. Although direct and indirect contact contamination are no less likely in conventional culture there is limited emphasis in recent literature on the occurrence and control of this problem. The extent of the problem of laboratory cross contamination is difficult to determine. Recognised incidents are generally not reported and it is likely that many if not most incidents are not recognised since sporadic contamination is unlikely to be suspected when it results in the isolation of a common organism from a specific source (e.g. *S. aureus *from a wound swab or *Salmonella enterica *from uncooked pork). Contamination is more likely to be considered when an organism is isolated from an uncommon source and when detailed typing of isolates of a specific species allows recognition of relationships not otherwise detected. This report suggests that laboratory cross contamination with *Salmonella *is not rare, particularly in food laboratories. Contamination with the laboratory positive control strain accounted for the majority of recognised false positive isolations in this study. Discussions with our client laboratories showed a variety of positive control strains were used including *S*. Alachua, *S*. Poona, *S*. Salford and *S*. Typhimurium. For practical purposes positive control strains should have an easily detectable phenotypic marker. The Oxoid manual recommends *S*. Typhimurium ATCC 14028 for the quality control of selenite broth and XLD agar and *S*. Poona NCTC 4840 for the quality control of bismuth sulphite agar [12]. The use of these strains as laboratory positive controls should not be recommended. *S*. Typhimurium is commonly isolated from many animal sources and is the second most common serotype isolated from humans worldwide [13]. *S*. Poona, although not as common a human pathogen as *S*. Typhimurium, has been associated with outbreaks and infections linked to reptiles [14] and cantaloupes [15].

The Health Protection Agency in the UK recommends the use of *Salmonella *Nottingham NCTC 7382 (16:d:e, n, z15) as a control strain [16]. *S*. Nottingham is an extremely rare serovar so if it is isolated contamination would immediately be suspected.

While our report deals specifically with *Salmonella enterica *there is no reason to believe that the problem is peculiar to this species. The risk of unrecognised cross contamination is probably greatest when the isolation process involved an enrichment step in a broth. This is a standard element in most procedures for isolation of bacteria from food. Cross contamination of solid media may be suspected on the basis that there is only one or a small number of colonies on the plate or the colonies may not be distributed in the expected way given the pattern of inoculation of the plate. There are no such visual clues from broth contamination.

It is apparent that cross contamination is also a significant problem with *M. tuberculosis*. Criteria for definition of a false positive *M. tuberculosis *incident have been published [7] although have not been universally accepted [17]. It is reasonable to suppose that there is also a risk of cross contamination with broth cultures of other species of bacteria.

Cross contamination should be suspected when a test isolate is indistinguishable from the laboratory positive control strain or a proficiency test isolate. Contamination should also be suspected if *Salmonella *is isolated from a specimen type which is rarely positive for that species/group of organism. Laboratories need to be aware that a false positive due to contamination does not always occur in an obvious time frame or sequence with a recent positive culture. There may be number of negative samples between the true positive culture and associated cross contaminated specimens. This has regularly been observed with *M. tuberculosis *contamination [5]. A study in Finland associated the use of automatic pipettes with an increased rate of *Salmonella *contamination in the laboratory [9]. However in our discussion with laboratories new staff and mislabelling of broths and plates were the commonly identified explanations for cross contamination.

## Conclusion

Standard laboratory precautions and routine hygiene and staff training are clearly important in reducing the risk of cross contamination but these measures may not be sufficient. In our laboratory we perform routine environmental monitoring for *Salmonella *to ensure that cleaning is of the required standard.

We suggest the following additional measures should be considered. Positive control strains should be processed and incubated in different areas from the test samples. With respect to food laboratories we suggest that specimens that are rarely positive for *Salmonella *(e.g. ready to eat foods and processed dairy products) should be processed at separate times, with separate equipment and if possible in separate rooms or benches from specimens that are relatively commonly positive for *Salmonella *(e.g. uncooked pork). We consider that broth cultures represent a particularly high risk for cross contamination of other media or the environment and therefore broth cultures should be sub-cultured to solid media in a designated area demarcated from areas where primary cultures are inoculated and if pipettors are used these should be dedicated to broth subculture. Use of aerosol resistant pipettor tips may be a useful additional precaution [9]. Manufacturers submitting samples of products for testing for *Salmonella *or other pathogens would be wise to retain a sample for each lot/batch tested for retest in the event of an unexpected positive result particularly in the case of products where a positive may lead to product recall and adverse publicity.

## Methods

### Isolates

Between 2000 and 2007 the National *Salmonella *Reference Laboratory (Ireland) received 7733 isolates of *Salmonella enterica *for typing. Isolates were from both human (n = 3687) and animal/food (n = 4046) sources.

### Serotyping

*Salmonella *isolates were assigned serotypes according to the Kauffmann-Whyte typing scheme using slide agglutination with standard antisera (Sifin Institute, Berlin, Germany, Murex Biotech Ltd., Dartford, England, and Dade-Behring Gmbh, Marburg, Germany).

### Antimicrobial susceptibility testing

Antimicrobial susceptibility testing was performed according to the disk diffusion method of the Clinical and Laboratory Standards Institute [18] (CLSI). The following antimicrobial agents (disk contents indicated in parentheses) were tested: ampicillin (10 μg), chloramphenicol (30 μg), streptomycin (10 μg), sulfonamides (300 μg), tetracycline (30 μg), trimethoprim (5 μg), nalidixic acid (30 μg), kanamycin (30 μg), ciprofloxacin (5 μg), ceftazidime (30 μg), gentamicin (10 μg) and minocycline (30 μg) (OXOID, Hampshire, United Kingdom). *Escherichia coli *ATCC 25922 was used as the control.

### Phage typing

Phage typing of *S*. Typhimurium and *S*. Enteritidis isolates was performed in accordance with the methods of the Laboratory of Enteric Pathogens, Health Protection Agency, Colindale, London, United Kingdom [19,20].

### Pulsed field gel electrophoresis

Pulsed field gel electrophoresis (PFGE) using the PulseNet standard protocol [21] was performed on selected isolates. DNA was digested using restriction enzymes XbaI (Roche, Basel, Switzerland) and BlnI (*Sigma*-Aldrich, Dorset, England) and DNA fragments were separated using the CHEF Mapper XA (Bio-Rad, California) system.

### Multi-locus variance analysis

Multi-locus variable-number tandem-repeats analysis (MLVA) using the method of Linstedt *et al*. [22] was performed on selected *S*. Typhimurium isolates. DNA was extracted using Qiaqen QIAamp mini kit (Qiagen, West Sussex, UK) and PCR was performed with flouresent primers (Sigma-Genosys, Suffolk, UK) using Qiagen Multiplex PCR master mix kit (Qiagen) on a GeneAmp PCR system 9700 thermal cycler (Applied Biosystems, Chesire, UK). Fragments were separated using a Beckman Coulter CEQ™ 8000 DNA analysis system (Beckmann-Coulter, Fullerton, CA).

### Review of records

The collection of isolates and our records were reviewed to identify possible episodes of laboratory cross contamination and sending laboratories were contacted to request submission of quality control strains (where not previously submitted) and to discuss the possibility of cross contamination.

## Authors' contributions

ND and MC conceived of and participated in the design of the study. ND drafted the manuscript. ND, JOC, GMD and GD carried out the serotyping, AST, PFGE and VNTR. MC helped to draft the manuscript. All authors read and approved the final manuscript.

## Supplementary Material

Additional file 1 **Summary of all Suspected Contamination Incidents investigated by NSRL from 2000–2007**. The table provided represents all the suspected contamination incidents investigated by the NSRL from the years 2000–2007, including the isolates concerned, their stated source and their probable cause. Click here for file

## References

[B1] Millar BC Xu J Moore JE Risk assessment models and contamination management: implications for broad-range ribosomal DNA PCR as a diagnostic tool in medical bacteriology J Clin Microbiol 2002 40 5 1575 1580 11980924 10.1128/JCM.40.5.1575-1580.2002 PMC130933

[B2] Caplan J Cleaning up Peter Pan's Mess Time 2007 http://www.time.com/time/business/article/0,8599,1593051,00.html

[B3] Laurel VL Meier PA Astorga A Dolan D Brockett R Rinaldi MG Pseudoepidemic of Aspergillus niger infections traced to specimen contamination in the microbiology laboratory J Clin Microbiol 1999 37 5 1612 1616 10203538 10.1128/jcm.37.5.1612-1616.1999 PMC84851

[B4] Katz KC McGeer A Low DE Willey BM Laboratory contamination of specimens with quality control strains of vancomycin-resistant enterococci in Ontario J Clin Microbiol 2002 40 7 2686 2688 12089309 10.1128/JCM.40.7.2686-2688.2002 PMC120566

[B5] Gascoyne-Binzi DM Barlow RE Frothingham R Robinson G Collyns TA Gelletlie R Hawkey PM Rapid identification of laboratory contamination with Mycobacterium tuberculosis using variable number tandem repeat analysis J Clin Microbiol 2001 39 1 69 74 11136751 10.1128/JCM.39.1.69-74.2001 PMC87682

[B6] Burman WJ Stone BL Reves RR Wilson ML Yang Z El-Hajj H Bates JH Cave MD The incidence of false-positive cultures for Mycobacterium tuberculosis Am J Respir Crit Care Med 1997 155 1 321 326 9001331 10.1164/ajrccm.155.1.9001331

[B7] de Boer AS Blommerde B de Haas PE Sebek MM Lambregts-van Weezenbeek KS Dessens M van Soolingen D False-positive mycobacterium tuberculosis cultures in 44 laboratories in The Netherlands (1993 to 2000): incidence, risk factors, and consequences J Clin Microbiol 2002 40 11 4004 4009 12409366 10.1128/JCM.40.11.4004-4009.2002 PMC139647

[B8] Wurtz R Demarais P Trainor W McAuley J Kocka F Mosher L Dietrich S Specimen contamination in mycobacteriology laboratory detected by pseudo-outbreak of multidrug-resistant tuberculosis: analysis by routine epidemiology and confirmation by molecular technique J Clin Microbiol 1996 34 4 1017 1019 8815074 10.1128/jcm.34.4.1017-1019.1996 PMC228944

[B9] Pelkonen SaK H Estimating causes and rate of laboratory contamination International Symposium Salmonella and Salmonellosis 2006 555 556

[B10] McNicholas S Morrisey M Glancy J Coleman A Corbett-Feeney G Cormican M Pseudo Hospital Acquired Salmonellosis associated with Laboratory Cross-Contamination Irish Journal Of Medical Science 2004 1115452945

[B11] Mossong J Marques P Ragimbeau C Huberty-Krau P Losch S Meyer G Moris G Strottner C Rabsch W Schneider F Outbreaks of Monophasic Salmonella enterica Serovar 4,[5],12:i:- in Luxembourg, 2006 Euro Surveill 2007 12 6 E11 E12 17991400 10.2807/esm.12.06.00719-en

[B12] Oxoid Oxoid Manual 2006 http://www.oxoid.com/UK/blue/prod_detail/prod_detail.asp?pr=CM0469&c=UK&lang=EN

[B13] WHO Global Salm Surv Progress Report http://www.who.int/salmsurv/links/GSSProgressReport2005.pdf

[B14] Anon Baby dies of Salmonella poona infection linked to pet reptile Commun Dis Rep CDR Wkly 2000 10 18 161 10842454

[B15] Anon Multistate outbreak of Salmonella poona infections – United States and Canada, 1991 MMWR Morb Mortal Wkly Rep 1991 40 32 549 552 1861671

[B16] Anon An Update for Participants Food EQA News 2005 2 1

[B17] Carroll NM Richardson M van Helden PD Criteria for identification of cross-contamination of cultures of Mycobacterium tuberculosis in routine microbiology laboratories J Clin Microbiol 2003 41 5 2269 author reply 2269–2270. 12734301 10.1128/JCM.41.5.2269-2270.2003 PMC154733

[B18] NCCLS Performance standards for antimicrobial disk susceptibility tests Approved standard, NCCLS document M2-A8 2003 8 NCCLS, Wayne, Pa

[B19] Ward LR de Sa JD Rowe B A phage-typing scheme for Salmonella enteritidis Epidemiol Infect 1987 99 2 291 294 3315705 10.1017/s0950268800067765 PMC2249269

[B20] Anderson ES Ward LR Saxe MJ de Sa JD Bacteriophage-typing designations of Salmonella typhimurium J Hyg (Lond) 1977 78 2 297 300 321679 10.1017/s0022172400056187 PMC2129838

[B21] Ribot EM Fair MA Gautom R Cameron DN Hunter SB Swaminathan B Barrett TJ Standardization of pulsed-field gel electrophoresis protocols for the subtyping of Escherichia coli O157:H7, Salmonella, and Shigella for PulseNet Foodborne Pathog Dis 2006 3 1 59 67 10.1089/fpd.2006.3.59 16602980

[B22] Lindstedt BA Vardund T Aas L Kapperud G Multiple-locus variable-number tandem-repeats analysis of Salmonella enterica subsp. enterica serovar Typhimurium using PCR multiplexing and multicolor capillary electrophoresis J Microbiol Methods 2004 59 2 163 172 10.1016/j.mimet.2004.06.014 15369852

