# Supplementary material for: Role of subtyping in detecting Salmonella cross contamination in the laboratory
Source: BMC Microbiol. 2009 Jul 31;9:155. doi: 10.1186/1471-2180-9-155 (PMC2727516; doi:10.1186/1471-2180-9-155)
Supplement: Additional file 1 — Summary of all Suspected Contamination Incidents investigated by NSRL from 2000–2007. The table provided represents all the suspected contamination incidents investigated by the NSRL from the years 2000–2007, including the isolates concerned, their stated source and their probable cause. [file 1471-2180-9-155-S1.doc]

## Additional Files Table 1 - Summary of all Suspected Contamination Incidents investigated by NSRL from 2000-2007

##

| **Lab** | **Year** | **Strain** | **Isolate no** | **Source** | **Cause** |
| --- | --- | --- | --- | --- | --- |
|  |  |  |  |  |  |
| A | 2001 | *S*. Typhimurium DT4 | 01-1181 | Mussels | ? Test |
|  |  |  | 01-1182 | Mussels |  |
|  |  |  | 01-1183 | Mussels |  |
|  |  |  | 01-1184 | Mussels |  |
|  |  |  | 01-0852 | Beef |  |
|  |  |  | 01-0944 | Pork |  |
| A | 2002 | *S*. Typhimurium DT4 | 02-0318 | Beef | ? Test |
|  |  |  | 02-0319 | Beef |  |
|  |  |  | 02-0378 | Beef |  |
|  |  |  | 02-0423 | Beef |  |
|  |  |  | 02-0868 | Beef |  |
|  |  |  | 02-0891 | Crab |  |
|  |  |  | 02-0892 | Crab |  |
|  |  |  | 02-0231 | Mackerel |  |
|  |  |  | 02-0321 | Sausage |  |
| A | 2003 | *S*. Typhimurium DT4 | 03-0099 | Not stated | ? Test |
| A | 2003 | *S*. Typhimurium DT132 | 03-1003 | Beef | PC |
|  |  |  | 03-1081 | Beef |  |
|  |  |  | 03-1083 | Beef |  |
|  |  |  | 03-1085 | Beef |  |
|  |  |  | 03-1086 | Beef |  |
|  |  |  | 03-1091 | Drain swab |  |
|  |  |  | 03-1197 | Pork |  |
| A | 2004 | *S*. Typhimurium DT132 | 04-0062 | Beef | PC |
|  |  |  | 04-0096 | Powder |  |
|  |  |  | 04-0097 | Pork |  |
|  |  |  | 04-0216 | Burger |  |
| B | 2002 | *S*. Panama | 02-0505 | Dairy product | PC |
|  |  |  | 02-0506 | Dairy product |  |
| C | 2003 | *S*. Enteritidis PT1 | 03-0537 | Human stool | Test |
| D | 2003 | *S*. Chandans | 03-0398 | Dairy product | PT |
| D | 2007 | *S*. Typhimurium, Untypable * | 07-0071 | Mussels | PC |
|  |  |  | 07-0072 | Mussels |  |
|  |  |  | 07-0461 | Fish |  |
| E | 2004 | *S*. Poona | 04-0439 | Not stated | PC |
|  |  |  | 04-0440 | Pork |  |
| E | 2004 | *S*. Typhimurium DT132 | 04-1112 | Pharmaceuticals | PC |
| E | 2005 | *S*. Typhimurium, Untypable | 05-0900 | Dairy product | Test (Swine) |
| E | 2007 | *S*. Typhimurium, Untypable | 07-0146 | Dairy product | Test (Swine) |
| F | 2004 | *S*. Goldcoast | 04-0678 | Food | PC |
| G | 2004 | *S*. Typhimurium DT132 | 04-0461 | Roast chicken | PC |
|  |  |  | 04-0462 | Roast chicken |  |
|  |  |  | 04-0517 | Contaminant |  |
| G | 2005 | *S*. Poona | 05-0781 | Frozen Herring | PC |
|  |  |  | 05-0783 | Frozen Herring |  |
| H | 2004 | *S*. Poona | 04-0065 | Poultry | PC |
|  |  |  | 04-0982 | Poultry |  |
|  |  |  | 04-1015 | Poultry |  |
| H | 2005 | *S*. Poona | 05-0296 | Poultry | PC |
|  |  |  | 05-0593 | Mussels |  |
| H | 2007 | *S*. Kentucky | 07-0677 | Fish factory swab | Test (Poultry) |
| H | 2007 | *S*. Poona | 07-0678 | Fish factory swab | PC |
| I | 2005 | *S*. Java | 05-0580 | Human stool | Test (Faeces) |
| J | 2005 | *S*. Poona | 05-0150 | Cheese | PC |
| K | 2006 | *S*. Mikawasima | 06-0565 | Human stool | Test (Faeces) |
|  |  |  | 06-0566 | Human stool |  |

PC = Positive Control

PT = Proficiency Test

Test (Swine/Poultry) = An isolate from this source isolated in the laboratory prior to the suspected contamination incident was the suspected source of contamination.

* The positive control isolate in this laboratory was *S*. Typhimurium ATCC14028 which typed as DT132. The O antigens of the test isolates were weakly expressed and did not react with the phage typing panel. However they shared the same MLVA profile as the positive control, i.e. 01-10-04-01-03.
